# *In-vitro* Antioxidant, Cytotoxic, Cholinesterase Inhibitory Activities and Anti-Genotoxic Effects of* Hypericum retusum *Aucher Flowers, Fruits and Seeds Methanol Extracts in Human Mononuclear Leukocytes

**Published:** 2017

**Authors:** Cumali Keskin, Necmettin Aktepe, Yunus Yükselten, Asuman Sunguroglu, Mehmet Boğa

**Affiliations:** a *Mardin Artuklu University, School of Health, Department of Nutrition and Dietetics, Mardin, Turkey.*; b *Mardin Artuklu University, School of Health, Department of Nursing, Mardin, Turkey*; c *Ankara University, School of Medicine, Department of Medical Biology, Ankara, Turkey*; d *Dicle University, Faculty of Pharmacology, Department of Pharmasotic Technology, Diyarbakır, Turkey.*

**Keywords:** *Hypericum retusum AUCHER*, Antioxidant, Cytotoxic, Anticholinesterase, Antigenotoxic

## Abstract

The present study investigates the antioxidant, anticancer, anticholinesterase, anti-genotoxic activities and phenolic contents of flower, fruit and seed methanol extracts of *Hypericum retusum *AUCHER. The amounts of protocatechuic acid, catechin, caffeic acid and syringic acid in methanol extracts were determined by HPLC. Total phenolic content of *H. retusum *seed extract was found more than fruit and flower extracts. The DPPH free radical scavenging activity of flower and seed methanol extracts showed close activity versus BHT as control. Among three extracts of *H. retusum *only flower methanol extract was exhibited considerable cytotoxic activities against to HeLa and NRK-52E cell lines. Moreover, seed methanol extract showed both acetyl and butyrl-cholinesterase inhibitory activity. The highest anti-genotoxic effects were seen 25 and 50 μg/mL concentrations. In this study, the extracts showed a strong antioxidant and anti-genotoxic effect. The seed extract was more efficient- than extracts of fruit and flowers. Our results suggest that the antioxidant and anti-genotoxic effects of extracts depend on their phenolic contents. Further studies should evaluate the *in-vitro* and *in-vivo *the benefits of *H. retusum* seed methanol extracts.

## Introduction

Free radicals can chemically relate with cell elements (DNA, lipid or protein) and steal their electrons so as to become resolute. Some of them return to reactive oxidants in biological system. Reactive oxygen species (ROS) have the potential to cause serious damage to the cell. They play a major role in the development of many chronic and neurodegenerative disorders, such as Parkinson’s and Alzheimer’s disease, heart and blood vessel disorders, muscle degeneration, gen mutation and cancer (1, 2). Hydrogen peroxide (H_2_O_2_) is one of the main ROS members that is known to provoke DNA damage in different cell types (3). Furthermore, caspase-3 is known as the most important stimulant of apoptosis and necrosis which are activated by H_2_O_2_ (4). 

Cancer is a general term for a category of diseases. Cancer cells grow abnormally and they frequently invade healthy cells in the body (5). Many external and internal reasons such as mutations, tobacco, anthropogenic activities, immune conditions, chemical substances, radiation can trigger the cancer process (6). Antioxidants defence systems may protect against these diseases. Many clinical trials proved that the daily consumption of nutritional supplements, food additives, phytochemicals and modified diets could prevent many cancer types (7). The phytochemicals are chemical compounds that are formed naturally by plants. Inflammatory processes and oncogenic transformation were found as biological targets of phytochemicals in mammalian cells (8). It was highlighted in recent epidemiological studies that routine consumption of specific phytochemicals in diet contributed to cancers (9). 

Natural products especially those originated from nature have been used for medical care of many types of illnesses for thousands of years. Medicinal plants have been used as medicines in China, Mesopotamia, India and Egypt from ancient times and a significant amount of modern effective drugs have been improved from medicinal plants (10). Natural products derivate from medicinal plants still serve as a rich source for the development of modern drugs for the treatment of cancer and other disease (11, 12). 

The genus Hypericum L. (St. John’s Wort, Hypericaceae), which contains 469 species, is involved in relatively dry temperate zones through the world, only excluding Antarctica. There are 96 *Hypericum* species in Turkey flora of which 46 are endemic (13, 14). Some *Hypericum species* are used as wound healing, sedatives, stomach diseases (gastritis, ulcer), icterus, haemorrhoids, cuts, burns and antipyretic in febrile illness (15). 

Many of bioactive secondary metabolites have been identified from different extracts of *Hypericum* species, including hypericin, pseudohypericin, quercetin, rutin, isoquercitrin, hyperoside, amentoflavone, hyperforin, adhyperforin, mangiferin and kielcorin (13). These compounds were isolated from different parts of *Hypericum* species by using several solvents. These compounds potentially display many biochemical activities such as antidepressant (especially *H. perforatum*), antioxidant, antimicrobial, anti-proliferative, anticholinesterase and cytotoxic activity (16-20). 

Investigations of *in-vitro* antioxidant, cytotoxic, anticholinesterase, anti-genotoxic effects, and individual phenolic compounds of fruit, flower and seed methanol extractions of *H. retusum* plant were aimed at this study.

## Experimental

*Instrumentation*

Spectrophotometric measurements were performed by using Shimadzu RF–1501 UV-VIS (ultraviolet-visible spectrophotometer). Anticancer (cytotoxic) activities of samples were spectrophotometrically measured by well known MTT assay (Microplate spectrophotometer system (BioTek® Epoch Microplate Spectrophotometer, Winooski-USA). Fluorescent Inverted Microscope (Olympus), Olympus CKX41 microscope, refrigerated centrifuge (Hettich Universal 30 RF), CO_2_ incubator (5% CO2, 95% humidity and 37 ºC) (Labotect) were used for Alkaline Comet Assay method.

*Collection of plant material*

Plants were sampled from Mardin-Ömerli road 4 Km (Zınnar valley) on flowering, fruiting and seedings stage from April to July. Plants materials were maintained at the Mardin Artuklu University Herbarium (2014-3-4-5-MAU). Plants were identified and authenticated by Dr. Cumali Keskin.

*Preparation of plant methanol extracts*

The 100 g plant materials were dehydrated in the shade (25 ± 2 °C) for 10 days. A total of 20 g of each material was grounded in a grinder with a 2 mm diameter mesh and incubated with 200 mL (99%) methanol at room temperature for 3 days. The obtained extracts were filtered with Whatman No.1 filter paper and methanol phase was removed on rotary evaporator under vacuum. Nearly, 2 g of the crude methanol extracts were obtained from each part of the plant material. The extracts were kept in dark and airtight glass bottles at -20 ºC until used for experimental studies. 

*Determination of total phenolic content*

Folin-Ciocalteu method applied to methanol extracts to determine their total phenolic contents (21). Absorbance of tested solutions was measured at 765 nm. Results were expressed as gallic acid equivalents (GAE) µg GAE/g extract of dry plant material) using the following equation obtained from a linear curve that gallic acid used a standard (y = 0.008X+0.004, R^2 ^= 0.9980).

*Chromatographic analyses*

The methanol extracts of different parts of H. *retusum* were analysed for their phenolic compounds by HPLC (Agilent 1260 Infinity HPLC-DAD with Chem Station revision B.04.01 software). Phenolic compounds were separated with the Agillent ZORBAX reverse phase C18 column (250 x 4,6- 5 μM) thermostated at 35 °C. Methanol and acetic acid–water (2:98 v/v) were used for gradient elution. Flowers, fruits, and seeds crude extracts (10 mg) were dissolved in methanol (10 mL) before injection to HPLC (20 μL). The phenolic compounds were monitored at 280 nm wavelength.

*Antioxidant Activity Assays (Reducing power activity)*

FRAP (ferric reducing antioxidant power) assay (22) was used to determine the antioxidant activities of plant methanol extract (10-500 mg in 1 mL of ethanol). The absorbance of prepared solutions was measured at 700 nm wavelength. The BHT (butylatedhydroxytoluen) and the BHA (butylatedhydroxyanisole) were used as standard antioxidants.

*DPPH Radical Scavenging Activity*

The free radical scavenging activity was quantitatively tested using 1,1-diphenyl-2-picryl-hydrazil (DPPH) based on the well-known procedure described in literature (20). The absorbance was measured at 517 nm wavelength. Following equation was applied to determine the percentages of inhibitions (Dorman, 2004). 

Inhibition %=A517 of control-A517 of sample A517 of controlx100

*Determination of Acetyl/Butyrylcholinesterase inhibitory activity*

The method described by Ellman, (23) was applied for cholinesterase inhibitory activities. *H. retusum *fruit flower and seed crude extracts were dissolved in ethanol to give concentrations of 4000 μg/mL. Aliquots of 150 mL of 100 mM sodium phosphate buffer (pH 8.0), 10 μL of sample solution and 20 μL AChE (or BChE) solution were mixed and incubated for 15 min at 25 ºC. Finally, 10 μL of DTNB (5,5-dithio-bis (2-nitrobenzoic acid) and 10 μL acetylthiocholine iodide (or butyryl thiocholine iodide) were added. The last concentrations of the mixture were 200 μg/mL. Inhibition percentages were calculated by using the following equation while galanthamine as a standard drug:

$\mathrm{Inhibition},\%=\left(\frac{A_{\mathrm{control}}-A_{\mathrm{sample}}}{A_{\mathrm{control}}}\right)\mathrm{x100}$

*Cell lines, culture treatments*

ATCC CCL-2 HeLa (human cervix cancer) and ATCC CRL-1571 NRK-52E lines (rat kidney epithelium cell) cultured by recommended protocols of manufacturers were applied to plants methanol extracts to determine their sensitivities by the MTT colorimetric assay. The cells were seeded at 10^4 ^cells/100 µL into each well of 96-well plates and incubated for 24 h at 37 °C and in 5% CO_2. _Next, the culture medium was abolished and the extracts were added to the wells in different concentrations. 

The exposure concentrations were expressed as µg/mL for the plant methanol extracts. Both of cell lines were exposed to plant extracts mentioned above.

*MTT cytotoxicity assay*

Among the enzyme-based assays, the MTT assay is the best known method to determine the mitochondrial dehydrogenase activities in the living cells. Cytotoxicity assays together with cell viability studies are used for drug screening of chemicals. The method described by Alley *et al*. (24) was applied for cytotoxic activity of plant methanol extracts. In all applied tests the absorbance was measured at 590 nm wavelength. LC_50 _values were calculated by using the following equation as the percentages of solvent controls; 

Inhibition, %=100-corrected mean Asamplecorrected mean Asolvent comtrolx100

*Statistical method*

All of the samples were subjected to methods in triplicate (n = 3). The results were presented as average ± SD, where SD was standard deviation. Student’s *t*-*test *was applied to compare results. A P-value of less than 0.05 was accepted as significant.

*Anti-genotoxic Activity*

*Separation and incubation of mononuclear leukocytes:*

20 mL heparinised blood sample was taken from healthy, non-smoking 26-year-old male volunteers. 5 mL Histopaque-1077 were added into four sterile tubes. Successively, 5 mL heparinised blood were added slowly. The tubes were centrifuged at 2100 rpm at 25 degrees for about 30 min. After centrifugation of the lymphocytes, which accumulate in the middle layer of the tube, they were taken into empty tubes with the help of a 1 mL pipette. In order to remove the Histopaque solution 5 mL 1 mol/L phosphate buffer saline (PBS) (pH 7.4) were added to samples before centrifugation at 1600 rpm at 25 degrees for 10 min. The upper layer was removed and the leukocyte pellet was obtained.

*Cell viability assay:*

Trypan blue stain was used for the evaluation of cell viability assay. The cells were trypsinized and collected from the culture flask. Then, they were mixed with an equal volume (1:1) of trypan blue. They were incubated for 5 min, as well. Stained and unstained cells were counted under the light microscope. 

*Preparation and incubation of the mononuclear leukocyte suspension*

Dulbecco’s modified Eagle’s medium (DMEM) was used for the culture of human mononuclear leukocyte cells. 10% fetal bovine serum (FBS) and 5 μg/mL gentamicin were added to DMEM. Then the prepared cell culture flasks in 5% CO_2_ atmosphere at 37 °C was dropped into 1, 2 and 3 h of incubation. 

The human mononuclear leukocyte cells were used without extract and H_2_O_2 _as negative control. 0.7 mM H_2_O_2_ treated group without extract administration was used as positive control. The cells were incubated with different concentrations of (10-100 μg/mL) *H. retusum *methanol extracts.

*Determination of DNA damage (Alkaline Comet Assay):*

The human mononuclear leukocyte DNA damage (hydrogen peroxide induced) was analysed by alkaline comet assay (25, 26). 80 μL of 0.7% low melting point agarose in PBS was mixed with 10 µL (around 20,000 cells) of cell suspension treated with different concentrations of *H. retusum* flower, fruit and seed methanol extract at 37 °C. 

## Results and discussion

This study reports phenolic contents and cytotoxic, *in-vitro *antioxidant, anticholinesterase and anti-genotoxic effects of flower, fruit and seed methanol extracts of *H. retusum *sampled in the different seasonal stages. The effect of primary antioxidant and some other biochemical activities from different plant sources are originated from various types of phenolic compounds. However, these effects are not always associated with presence of large amounts of certain phenolic compounds (27). The total phenolic contents of flower, fruit and seed methanol extracts of *H. retusum *were determined as 130.75, 115.8 and 158.25, respectively. 

In this study the antioxidant activities of different parts of *H. retusum *were determined by the reducing power activity and DPPH assay. The reducing power activity is related with antioxidant activity. Existence of reducer compounds in extract causes the conversion of the Fe^3+^/ferricyanide complex to the ferrous form (28).

**Table 1:** Reducing power activities and (%)DPPH free radical scavenging activities of different parts of *H. retusum* methanolic extracts and BHA, BHT as Control (n=3, mean±standard deviation

	**Reducing power activity**					**DPPH (%)**				
**Concentrations ** **(µg/mL)**	***H. retusum***			**Controls**		***H. retusum***			**Controls**	
	**Flower**	**Fruit**	**Seed**	**BHA**	**BHT**	**Flower**	**Fruit**	**Seed**	**BHA**	**BHT**
10	0.27 ± 0.01	0.27 ± 0.01	0.22 ± 0.01	0.2 ± 0.01	0.2 ± 0.01	22.2 ± 0.5	28.97 ± 1.5	22.98 ± 1.3	72.04 ± 0.42	82.3 ± 0.3
20	0.26 ± 0.01	0.29 ± 0.01	0.29 ± 0.02	0.4 ± 0.02	0.3 ± 0.02	25.6 ± 0.6	30.80 ± 4.6	29.31 ± 1.6	79.03 ± 0.4	83.4 ± 0.1
50	0.27 ± 0.02	0.31 ± 0.01	0.32 ± 0.01	0.7 ± 0.03	0.5 ± 0.02	33.00 ± 0.7	34.43 ± 3.4	40.07 ± 1.5	80.41 ± 0.3	85.4 ± 0.3
100	0.34 ± 0.03	0.37 ± 0.3	0.42 ± 0.01	0.9 ± 0.05	0.6 ± 0.05	44.1 ± 1.5	51.17 ± 1.3	56.98 ± 0.8	81.15 ± 0.4	86.8 ± 0.3
250	0.44 ± 0.04	0.49 ± 0.03	0.60 ± 0.02	1.2 ± 0.14	0.9 ± 0.07	61.5 ± 1.0	74.67 ± 1.5	79.18 ± 0.1	84.75 ± 0.2	89.3 ± 04
500	0.60 ± 0.03	0.68 ± 0.02	0.82 ± 0.04	1.6 ± 0.20	1.1 ± 0.15	83.7 ± 1.6	77.71 ± 0.9	83.13 ± 0.4	85.2 ± 0.6	96.2 ± 0.8

**Table 2:** Percent cell inhibition of *H. retusum* flower methanol extracts on HeLa and NRK-52E cell lines

**Conc. (** **µ** **g/mL)**	**% cell inhibition**	
	**HeLa**	**NRK-52E**
0.781	-	4.61
1.562	25.45	17.75
3.125	28.98	24.77
6.25	30.34	42.36
12.5	46.02	59.75
25	57.61	74.25
50	78.40	90.80

**Table 3:** Effect of *H. retusum* methanol extracts on the antigenotoxicity induced by H_2_O_2_ in human mononuclear leukocyte by using comet assay

***Hypericum retusum *** **methanol extracts ** **Arbitrary Unit (AU)**			
**Applied Concentration**	**Flower**	**Fruit**	**Seed**
**10 ** **µ** **g/mL Extract** **+0.7 mM H**_2_ **O**_2_	243.18 ± 21.10	257.61 ± 7.50	161.62 ± 6.10
**25** **µ** **g/mL Extract** **+0.7 mM H**_2_ **O**_2_	132.99 ± 8.32	177.75 ± 12.07	228.97 ± 15.70
**50** **µ** **g/mL Extract** **+0.7 mM H**_2_ **O**_2_	168.22 ± 9.44	160.77 ± 5.52	105.76 ± 2.40
**100** **µ** **g/mL Extract** **+0.7 mM H**_2_ **O**_2_	276.77 ± 22.04	264.80 ± 14.67	213.53 ± 1.85
**Control**	47.78 ± 3.80	20.44 ± 1.39	18.17 ± 1.04
**Positive control** **0.7 mM H**_2_ **O**_2_	319.89 ± 22.83	320.88 ± 2.59	313.15 ± 3.42

**Table 4:** Comprehensive biological evaluation in view of antioxidant, cytotoxic, anticholinesterase and antigenotoxic activities.

**Plant Sample**	**Fraction/Extract**	**Total phenolic contents**	**Inhibition (%) of DPPH**	**Reducing power activities**	**IC**_50_ ** (** **µ** **g/mL)** **in HeLa cells**	**IC**_50_ ** (** **µ** **g/mL)** **in NRK-52E cells**	**Inhibition %, AChE** **(μg/mL)**	**Inhibition %, BChE** **(μg/mL)**	**Antigenotoxicity induced by H**_2_ **O**_2_ ** in human mononuclear leukocyte**	**Ref.**
*H. perforatum* L.	Aerial parts (Ethyl acetate extract)	355.01±0.4 mg g^-1^ extract	89.80				49.54	50.79		^42^
*H. hookerianum*	Leaves (Methanol extracts)	280 mg/g of dry mass	90.24	0.84	113.05^a^					^43^
*H. perforatum* L.	Aerial parts (Ethanol extracts)	35.35 ± 4.5 mg GAL/g	55.4	2.09	37^b^ (150mg/ml)					^44^
*H. amblysepalum*	Flower, fruit, seed (Methanol extracts)	115.8±1.4 μg 135.8±0.9 μg 154.5 ± 0.8 μg GAE/mg extract	89.07 83.17 84.13	0.62 0.66 0.63	NA 4.12 11.67	NA 4.39 2.86	NA NA 20.39	53.67 38.63 76.89		^20^
*Gracilaria tenustipitata*	Seaweeds (Aqueous extract)	94.94 ± 2.43 μg GAE/mg extract	63.37						53.6 ± 17.1 (Treatment dose 1 mg/ml) (Plasmid DNA)	^45^
*Casearia sylvestris* var. *Lingua*	Fruits (Hexane extract)				31.2^c^	18.2^d^				^5^
*H. retusum*	Flower, fruit, seed (Methanol extracts)	130.75±1.4 μg 115.8±0.9 μg 158.25±1.5 μg GAE/mg extract	83.7 77.71 83.13	0.60 0.68 0.82	17.66 NA NA	7.97 NA NA	NA NA 25.50	14.21 50.62 60.76	13.99 160.77 105.76 Arbitrary Unit (AU)	This study

a The human breast cancer cell line (MCF 7)

b The mouse breast cancer cell line (4T1)

c The human colon carcinoma (HCT-8)

d MDA-MB-435 (melanome) tumor cell lines

**Figure 1a:**
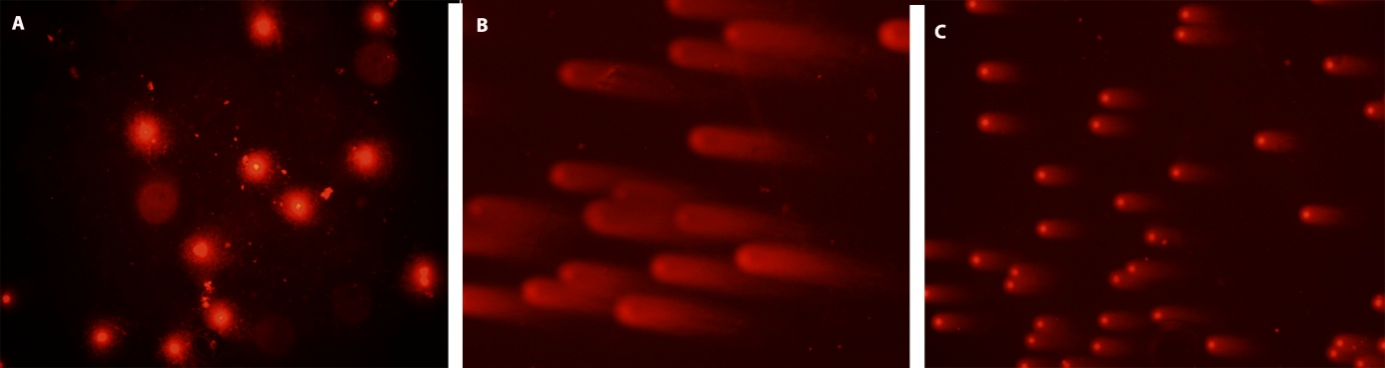
DNA damage visual classification, according to the relative proportion of DNA in the tail (cells between 0 and 4), provided from single-cell gel electrophoresis. “0” is undamaged cell, and “4” is the most heavily damaged cell. (a) Control cells, (b) positive control cells; treated only with 0.7 mM H2O2, (c) 50 μg/ml HPE + 0.7 mM H_2_O_2_, H_2_O_2_, HRE: *Hypericum retusum *seed extract ;H_2_O_2_: Hydrogen peroxide

**Figure 1b:**
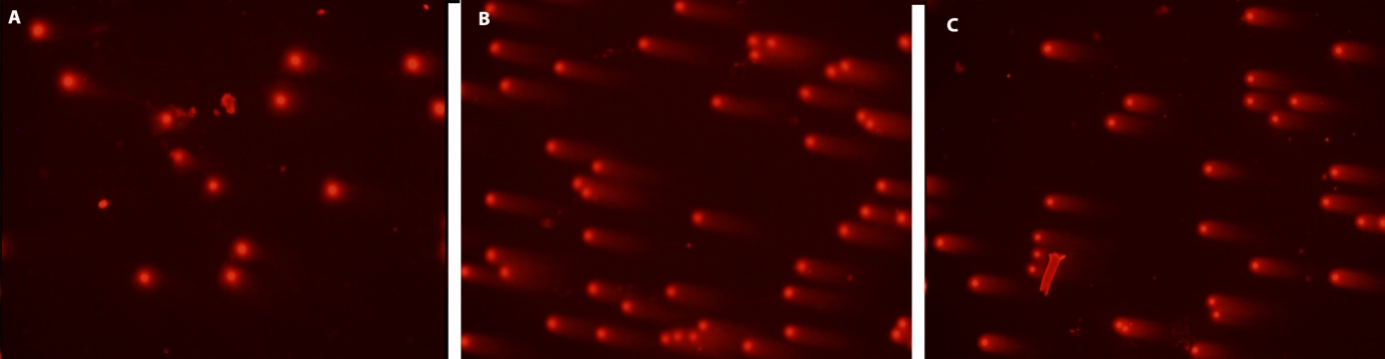
DNA damage visual classification, according to the relative proportion of DNA in the tail (cells between 0 and 4), provided from single-cell gel electrophoresis. “0” is undamaged cell, and “4” is the most heavily damaged cell. (a) Control cells, (b) positive control cells; treated only with 0.7 mM H_2_O_2_, (c) 50 μg/ml HRE + 0.7 mM H_2_O_2_, H_2_O_2_, HRE: Hypericum retusum fruit extract ; H_2_O_2_: Hydrogen peroxide

**Figure 2:**
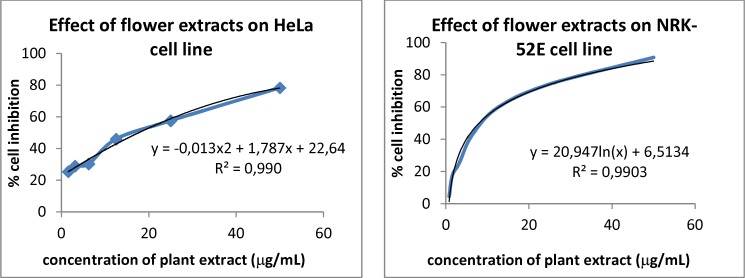
Effect of H. retusum flower methanol extract on HeLa and NRK-52E cell line

**Figure 3:**
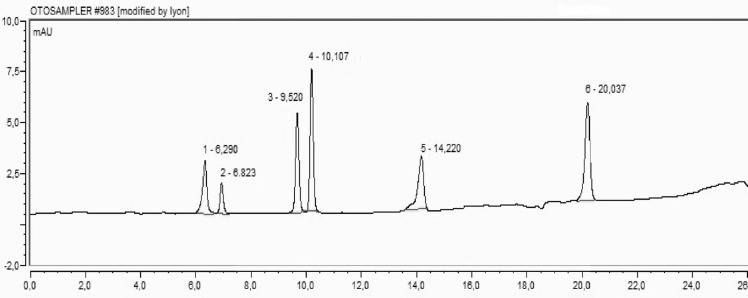
A representative chromatogram of stadard mixture of phenolic compounds. 1: Protocatechuic (t_R_:6.290), 2: Catechin (t_R_: 6.823), 3: Cafeic acid (t t_R_: 9.520), 4: Syringic (t_R_: 10.107), 5: p-coumaric acid(t_R_:14.220), 6: o-coumaric acid (t_R_: 20.037

So, the presence of reducing agents in *H. retusum *extracts causes the reduction of the Fe^3+^/ferricyanide complex to the ferrous form. Fe^3+ ^reduction is often used as an indicator of electron providing activity. The intensity of colour resulting from reducing of ferric ions depends on the reducing potential of the compounds present in the extracts. The intensity of the colour reflects the absorption, which is correlated to antioxidant activity (29). The reducing power activities of controls (BHA and BHT) and *H. retusum* flower, fruit and seed methanol extracts are demonstrated in detail Table 1. It is clear to conclude that reducing power activities reduced with the sequence of seed, fruit and flower extract. The reducing power activities of *H. retusum* methanol extracts were found lower than the controls. 

Inhibition (%) of DPPH free radical scavenging activity of methanolic extracts and controls were presented in Table 1. Decrease in the concentration of DPPH radicals due to the scavenging ability of the antioxidant contents of the *H. retusum* extracts. Linear increase in activities was observed with increasing concentrations. While the fruit methanol extracts showed a higher activity at lower concentration, the flower methanol extract showed more activity at higher concentrations. 

Percent cell inhibition of seven different concentrations of *H. retusum* flower methanol extracts on HeLa and NRK-52E cell lines were given in Table 2. The percentage tested cell inhibition profiles were found to be concentration dependent (Figure 2). The maximum concentration used in present study was 50 μg/mL. Fruit and seed methanol extracts of *H. retusum *showed not any remarkable results. The LC_50 _values of flower were17.66 and 7.97 μg/mL versus HeLa and NRK-52E cell line respectively. It was noted from Table 2 that a gradual increase in percentage inhibition was observed in all the cases.

Overall, it can be stated that *H. retusum *may contain a range of phytochemical constituents of anticancer compounds in the methanolic extracts (30). Synergistic effects of different bioactive compounds in the extract may be responsible for cytotoxic compounds (31). The anticancer activity of the flower extract on studied cells is explicit and the MTT assay suggests a mitochondrial relation.

No activity for flower and fruit extracts were observed but the seed methanol extracts showed 25.50 ± 0.74 % inhibitor activity against the AChE. However, all extracts showed inhibitory activity against to the BChE. Whereas the highest BChE inhibitory activity was observed in seed methanol extract, the lowest activity was observed in fruit methanol extract. The seed methanol extract showed the moderate inhibition against butyrylcholinesterase (60.76 ± 0.31%) as compared with galanthamine (81.83 ± 0.26%), at 200 mg/mL concentration. The dissimilarity behaviour of *H. retusum* methanol extracts against AChE and BChE inhibition could be attributed to their individual bioactivities. 

HPLC was used for the determination of protocatechuic acid (PCA), catechin, caffeic acid and syringic acid, p-qumaric acid and o-qumaric acid concentrations in fractional extracts of *H. retusum*. Standard mixture of phenolic compounds and their retention time chromatogram was given in Figure 3. The concentrations of selected compounds were varied in flower, fruit and seed methanol extracts of *H. retusum*. The protocatechuic acid levels of extracts were found as 17.93, 8.80 and 7.08 mg/Kg respectively. PCA has structural resemblance with other antioxidant compounds like as, gallic acid, caffeic acid, vanillic acid, and syringic acid. PCA has been found convenient for treatment and/or prophylaxis for a large number of various disorders associated with oxidative stress damage in multiple body systems *in-vitro* and *in-vivo* (32). Catechin was only detected in seed methanol extract as 34.40 mg/Kg. Also, syringic acid was only detected in fruit methanol extract as 0.63 mg/Kg. Caffeic acid, p-qumaric acid and o-coumaric acid were not detectable levels in extract samples. Catechin is a flavan-3-ol, a type of natural plant secondary metabolite. The consumption of catechins induces antioxidant, antiviral and anticancer activities. Catechins can reduce blood pressure, regulated plasma sugar level and stimulate hepatic lipid metabolism to contribute to the prevention of various lifestyle-related diseases, especially obesity (33-37). 

Moreover, catechin has a strong free radical scavenger activity (38). Therefore, the catechin concentrations of *H. retusum* seed methanol extracts authenticated the DPPH free radical of its scavenging activities. Syringic acid is a phenolic compound and it acts pharmacologically as an antioxidant to clear free radicals. Syringic acid can prevent diabetic cataract pathogenesis by inhibiting aldehyde reductase activity (39). Syringic acid showed beneficial effects in diabetic rats (40). In addition, syringic acid have promising potential for the prevention and control of human malignant melanoma (41). 

In this study, cell culture media contain human mononuclear leukocyte cells, the most powerful preventive effects of the methanol extracts of *H. retusum* flowers, fruits and seeds against H_2_O_2_ induce DNA damage with a single cell alkaline gel electrophoresis. In the prepared cell culture flask 1, 2 and 3 h mononuclear leukocyte DNA damage levels were analysed with Comet Assay method. The density of the fluorescence in the comet tail was scored as 0 (undamaged), 1, 2, 3, or 4 (maximal damage); as a result, the total score of each slide varied between 0 and 400 arbitrary units (AU). Photomicrographs of representative samples were depicted in ure 1a, b. The highest anti-genotoxic effects were seen between 10 and 100 µg/mL concentrations. At the concentrations below and above these levels anti-genotoxic effects were decreased. Anti-genotoxic effects of all applied concentrations of *H. retusum *flower, fruit and seed methanol extracts and positive and negative controls were given as Arbitrary Unit (AU) in Table 3. 

While the highest anti-genotoxic effects were observed at 25 µg/mL concentrations for *H. retusum* flower extract (132 AU), the highest anti-genotoxic effects were determined at 50 μg/mL concentrations for fruits (160 AU) and seeds extracts (105 AU). However, anti-genotoxic effect has been decreased at the above and below of this concentrations. Biological activity parameters were compared with literature and were presented in Table 4. 

## Conclusion

It is possible to conclude that *H. retusum *flower, fruit and seed methanol extracts presented comparable activity in view of total phenolic, antioxidant, cytotoxic (against HeLa and NRK-52E cells), cholinesterase inhibition (AChE and BChE) and anti-genotoxic activity. 

The present results show that the *Hypericum retusum* seed can be a rich source of natural antioxidants and may protect cells from some degenerative diseases and becoming cancer. The cytotoxic activity was achived from MTT assay test results, which were an indicator of the possible applicability of the extracts in further *in-vitro *and *in-vivo *studies. Furthermore, the researches should investigate the isolation, structure elucidation and identification of the bioactive compounds and the mechanism of actions of these extracts. By considering the cholinesterase inhibitory activities of extracts, it could be concluded that using these extracts as alternative medicine contributes to the management of central nervous system disorders such as Alzheimer’s disease or multiple sclerosis. The HPLC analysis in view of protocatechuic acid, catechin and syringic acid confirmed the biological activities of extracts. 

The present results suggest that *H. retusum *flower, fruit and seed extracts have a strong anti-genotoxic effect and this effect is more powerful in the seed extract than in the fruit and flower. It has been thought that the anti-genotoxic effect of extracts depends on their phenolic contents. Further studies should evaluate the *in-vitro* and *in-vivo* benefits of *H. retusum* seed methanol extracts.
